# Is there an androgen level threshold for aneuploidy risk in infertile women?

**DOI:** 10.1186/s12958-015-0034-z

**Published:** 2015-05-06

**Authors:** Norbert Gleicher, David H McCulloh, Vitaly A Kushnir, Nandita Ganguly, David H Barad, Kara N Goldman, Mark M Kushnir, David F Albertini, James A Grifo

**Affiliations:** The Center for Human Reproduction, New York, New York 10021 USA; The Foundation for Reproductive Medicine, New York, New York 10021 USA; New York University Fertility Center, New York University School of Medicine, New York, New York 10016 USA; ARUP Institute for Clinical and Experimental Pathology, University of Utah School of Medicine, Salt Lake City, UT USA; Department of Pathology, University of Utah School of Medicine, Salt Lake City, Utah USA

**Keywords:** Androgens, Aneuploidy, Preimplantation genetic diagnosis (PGS), in vitro fertilization (IVF)

## Abstract

**Background:**

Low functional ovarian reserve (LFOR) has been associated with hypoandrogenemia and increased embryo aneuploidy, while androgen supplementation has been reported to improve aneuploidy rates. We, therefore, assessed whether in infertile women undergoing in vitro fertilization (IVF) androgen concentrations are associated with aneuploidy rates.

**Methods:**

This study was performed in 2 academically affiliated fertility centers in New York City and an academically affiliated steroid chemistry laboratory in Utah. Androgen concentrations were measured in blinded fashion from 84 infertile women (age 40.3 +/− 2.4 years) at New York University (NYU), using a validated LC-MS/MS method, in cryopreserved serum samples of patients who had undergone IVF with concomitant preimplantation genetic screening (PGS), utilizing a 24-chromosome platform. The Center for Human Reproduction (CHR) provided plasma samples of 100 historical controls (ages 38.6+/−5.0 years) undergoing IVF without PGS. Statistical comparisons were made of androgen concentrations, and of associations between androgen concentrations and embryo aneuploidy.

**Results:**

Women undergoing IVF + PGS at NYU revealed no association between embryo aneuploidy and androgen concentrations but demonstrated significantly lower androgen concentrations than the 100 control patients from CHR,

**Conclusions:**

Though this study revealed no association between androgen levels and embryo ploidy, the extremely low androgen levels in the NYU study group raise the possibility of a threshold effect below which testosterone no longer affects aneuploidy. Before an androgen effect on embryo ploidy can be completely ruled out, a patient population with more normal androgen levels has to be investigated.

## Background

Aneuploidy represents a frequent phenomenon in human reproduction, and is to a large degree oocyte-dependent [1,2]. Though even young women at peak fertility produce a large number of aneuploid embryos, rates of aneuploidy increase with advancing female age [3]. Recent animal [4] and human data [5] suggest that loss of functional ovarian reserve (FOR), in itself, may be associated with increased embryo aneuploidy. Whether increases in aneuploidy are exclusively age-related, independently also associated with premature loss of FOR in association with premature ovarian aging (POA) or with both has, however, remained controversial. Some studies have suggested that POA, if adjusted for age, does not predispose to higher aneuploidy rates [6].

What causes the high rate of aneuploidy in human oocytes and embryos is so far only partially understood [1]. Androgen supplementation has, however, been suggested to reduce aneuploidy rates [7]. This observation is further supported by significant decreases in miscarriage rates with androgen supplementation, especially in older women [8]. Whether caused by physiologic female aging or POA, low functional ovarian reserve (LFOR) has, almost universally, also been associated with relative hypoandrogenemia [9].

These associations raise the interesting question as to whether androgen levels may be associated with aneuploidy risk? Here presented is a collaborative study between two New York City - based in vitro fertilization (IVF) centers attempting to answer this question.

## Methods

The Institutional Review Boards of New York University (NYU) School of Medicine and the Center for Human Reproduction – New York approved this study.

### Study population

Women were identified who presented to NYU between 2013 and 2014 for IVF with 24-chromosome pre-implantation genetic screening (PGS). Exclusion criteria were: hormonal suppression prior to cycle start [oral contraceptives, gonadotropin releasing hormone agonist (GnRH) down-regulation or GnRH antagonist], uses of donor oocytes, or if cryopreserved serum was unavailable.

Once the androgen concentrations of the study group were recognized to be low, the question arose whether these concentrations were typical of infertile women of corresponding age. To answer this question, we randomly selected plasma samples from 100 infertile women as controls, who had undergone IVF at the Center for Human Reproduction (CHR) during the same time as the NYU study group.

### Study parameters in IVF cycles

Cycle parameters analyzed included patient age at the time of oocyte retrieval, day 2 follicle stimulating hormone (FSH) and estradiol (E2) levels, anti-Müllerian hormone (AMH), total units of gonadotropin [FSH and human menopausal gonadotropins (hMG)], number of days to human chorionic gonadotropin (hCG) trigger, E2 level on the day of ovulation trigger, numbers of oocytes and metaphase-II (M-II) oocytes retrieved, number of two pronuclear (2PN) zygotes, number of euploid and aneuploid and euploid blastocysts, and embryos with no diagnosis. A euploid-index was calculated by dividing the total number of euploid embryos by the total number of embryos biopsied (euploid + aneuploid + no diagnosis). Serum samples were assayed for dehydroepiandrosterone (DHEA), Androstenedion and total testosterone.

### Ovarian stimulation

Before initiation of treatment, menstrual day 2 or 3 serum E2 and FSH levels were assessed. Patients with acceptable parameters (E2 < 75 and FSH <13.5) were stimulated using injectable gonadotropins (Follitropin beta, Schering Plough, NJ; Serono Pharmaceuticals, Rockland, MA; Menotropins, Ferring Pharmaceuticals, Parsippany, NJ), with LH suppression achieved using a GnRH antagonist (ganirelix acetate, Organon; cetrorelix, Serono). Ovulation was triggered when ≥ 2 follicles reached ≥ 17 mm in diameter, and ultrasound-guided trans-vaginal oocyte retrieval was performed 34–36 hours later.

### Laboratory assays

#### Preimplantation genetic diagnosis (PGS)

Laser-assisted breaching of the zona pellucida was performed on day 3 (Cronus, Research Instruments, UK). Embryos were assessed on days 5 and 6, and fully differentiated blastocysts meeting criteria underwent trophectoderm biopsy. The trophectoderm cells extruding from the expanded blastocyst were gently pulled using suction, and laser was used to remove cells at cell junctions without disrupting the inner cell mass. Biopsied trophectoderm cells were loaded into PCR tubes and sent to the reference laboratory for 24-chromosome analysis using array comparative genomic hybridization (aCGH) as previously described [10,11]. Following biopsy, blastocysts were vitrified to be replaced in subsequent frozen cycles.

#### Androgen assays

Plasma samples of IVF patients were stored at −80°C at NYU since cycle start. The samples were de-identified and shipped on dry ice to the ARUP Institute (ARUP Institute Laboratories, Salt Lake City, Utah), where they were analyzed using a validated liquid chromatography tandem mass spectrometry method (LC-MS/MS) for dehydroepiandrosterone (DHEA), Androstenedion, as well as total testosterone [12].

Androgen concentrations of the CHR control group were also assessed using LC-MS/MS, though at a commercial laboratory (LabCorp, Burlington, North Carolina). Two samples from each patient were analyzed, a baseline sample obtained at initial presentation to the center and a sample at IVF cycle start after on average 6–8 weeks of supplementation with DHEA, 25 mg TID (Fertinatal®, Fertility Nutraceuticals, LLC, New York, N.Y.) [13].

Five samples were investigated in both laboratories (Utah and Bulington). In all five samples inter-assay variability between the laboratories was <5%.

#### Reagents and standards

Standards of testosterone, Androstenedion and DHEA, were purchased from Cerilliant (Austin, TX); the internal standards were deuterium labeled analogs of the steroids, d_3_-testosterone (Sigma, St Louis, MO), d_7_-androstenedione (Ceriliant) and d_5_-DHEA (CDN Isotopes, Quebec, Canada). All other chemicals were of the highest purity commercially available.

#### Liquid chromatography tandem mass spectrometry

Plasma samples were analyzed for DHEA, Androstenedion and testosterone using liquid chromatography tandem mass spectrometry (LC-MS/MS) as previously described [12]. Briefly, steroids were extracted from samples, derivatized with hydroxylamine to form oxime derivatives, and analyzed on a triple quadruple mass spectrometer (AB5500, AB Sciex, Foster City, CA), using an electrospray ion source in positive ion mode. The HPLC system consisted of series 1260 HPLC pumps (Agilent Technologies), and an HTC PAL auto sampler (LEAP Technologies, NC), equipped with a fast wash station.

The quadruples Q1 and Q3 were tuned to unit resolution and the mass spectrometer conditions were optimized for maximum signal intensity of each steroid. Two mass transitions were monitored for each steroid and its internal standard. Quantitative data analysis was performed using software Analyst™ 1.5.2. Calibration curves were generated using six calibrators; three quality control samples were analyzed along with the samples.

Specificity of the analysis for each steroid in every sample was evaluated by comparing concentrations determined by using primary and secondary mass transitions of each steroid and its internal standard [14]. Limit of quantification (LOQ) was 0.01 ng/mL for testosterone and Androstenedion, and 0.05 ng/ml for DHEA; Intra-and inter-assay imprecisions were <8.0 percent.

### Statistical analysis

Statistical analysis was performed using IBM SPSS Statistics for Windows, Version 21.0. (Armonk, NY: IBM Corp).

Continuous variables are presented as mean ± standard deviation. If not normally distributed, the variables were log transformed, and concentrations were presented as mean and 95% confidence intervals (CI). Differences between continuous variables were, as appropriate, assessed using ANOVA or ANCOVA.

We used cluster analysis to characterize the relationships between the variables. A linear regression model was used to assess the relationship of the number of euploid embryos as a function of total of two-pronuclear (2PN) embryos produced per patient and number of aneuploid embryos. We then further used linear regression models to evaluate how the concentration of DHEA, Androstenedion and total testosterone on cycle day-2 might affect this model.

## Results

The NYU study group was comprised of 84 women between ages 35 and 45 years (mean, 40.3 ± 2.4) who underwent IVF with embryo culture to blastocyst stage and trophectoderm biopsy. Patient characteristics are presented in Table 1.

**Table 1:** **Patient characteristics in NYU study patients**

	**n**	**Mean**	**SD or 95% CI**
*Age (years)*	84	40.30	2.44
*Day-2 FSH (IU/mL)*	84	6.64	2.37
*Day-2 E2 (pg/mL)*	84	43.10*	38.82 - 47.77*
*AMH (ng/mL)*	69	1.77*	1.47 - 2.13*
*Total gonanadtropins (IU)*	83	4,098	1380
*FSH (IU)*	83	2,646	1138
*HMG (IU)*	83	1,451	889
*Days to hCG trigger*	83	9.55	1.59
*E2 on day of trigger (pg/mL)*	83	2282*	2066 - 2522*
*Number of oocytes*	84	13.35*	11.97 - 14.88*
*Number of MII oocytes*	84	11.85	6.13
*2PN oocytes*	84	7.76*	6.84 - 8.80*
*DHEA (ng/dL)*	84	294.7*	257.5 – 337.4*
*Androstenedione (ng/dL)*	84	60.1*	55.8 – 64.6*
*Total testosterone (ng/dL)*	84	16.7*	15.4 – 18.1*
*Number of euploid embryos*	84	1.51	1.81
*Number of aneuploid embryos*	84	3.62	2.93
*Embryos with no diagnosis*	84	0.31	1.01
*Ploidy Index*	**84**	**0.29**	**0.29**

*Denotes P<0.05.

### Functional ovarian reserve (FOR) assessment

Considering their ages, patients in this group demonstrated almost excessively good functional ovarian reserve for their age, defined by mean day-2 FSH of 6.6mIU/mL (95% CI, 6.1 – 7.2), mean estradiol of 43.1 pg/mL (95% CI, 38.8 – 47.8) and AMH of 1.8 ng/mL (95% CI, 1.5 - 2.1). They also produced exceptionally good oocyte yields for their ages, with a mean of 13.4 (95% CI, 12.0 - 14.9).

### Androgen concentrations

Cycle day-2 DHEA concentrations were widely distributed between 96.6 ng/dL and 865 ng/dL with [mean of 290.9 (95% CI, 261.2 to 324.0) ng/dL. Kushnir et al. reported the normal range for DHEA in post menarchal women to be 111–770 ng/dL with concentration declining by approximately 15 percent per decade of life [10]. Only three (3.6%) of women demonstrated DHEA below the lower cut-off level of 111 ng/dL.

When the DHEA concentrations of the NYU patient group, however, were compared to a randomly selected historical control group of 100 infertile women of similar age (Table 2), before and after supplementation with DHEA, undergoing IVF at CHR, DHEA concentrations of NYU patients were similar to CHR baseline levels [294.7 ng/dL; (95% CI, 257.5 – 337.4); P = 0.23] but highly significantly lower to CHR post-supplementation levels [595.2 ng/dL (95% CI 539.2 – 651.1); P < 0.0001).

**Table 2:** **Comparison of androgen levels between NYU study and CHR control group**

	**NYU-Baseline**		**CHR –Baseline**		**CHR post DHEA**	
	**(mean & 95% CI)**		**(mean & 95% CI)**		**(mean & 95% CI)**	
*DHEA (ng/dL)*	290.97	261.27 to 324.03^1^	294.733	257.46 to 337.40	595.21	539.28 to 651.14^1^
*Androstenedion (ng/dL)*	60.08	55.86 to 64.62^2^	78.98	70.56 to 88.47^2^	123.64	110.77 to 138.00^2^
*Testosterone (ng/dL)*	16.67	15.35 to 18.11^2^	21.77	19.60 to 24.18^2^	32.14	28.62 to 36.09^2^

^1^P < 0.0001; ^2^P < 0.001; The table demonstrates that DHEA levels were similar between NYU and CHR patients at treatment start but post-DHEA supplementation CHR levels were significantly higher. Androstenedion and testosterone levels were even at baseline lower in NYU than CHR patients.

Cycle day-2 Androstenedion in NYU patients ranged from 18.9 to 134 ng/dL [average 60.0 ng/dL ; (95% CI 56.0 – 67.1). Kushnir et al. reported normal levels of Androstenedion in 323 post-menarchal women to be 33–213 ng/dL, with concentrations also declining by approximately 5 percent per decade of life [10].

Among 100 randomly selected women at CHR pre-DHEA supplementation day-2 Androstenedion concentrations were significantly higher than those of NYU patients; 78.9 ng/dL (95% CI 70.5 - 88.5) (P < 0.001). After 6 weeks of DHEA supplementation, Androstenedion concentrations among CHR patients even rose further to 123.6 ng/dL (95% CI, 110.7 - 138.0; P < 0.001).

Cycle day-2 total testosterone among NYU women was 16.7 ng/dL (95% CI, 15.4 - 18.1). Kushnir et al. reported normal levels of total testosterone in 323 post-menarcheal women to be 9 – 55 ng/dL , with concentrations not being age-dependent throughout all reproductive ages [10]. Total testosterone levels appear more predictive of pregnancy than free testosterone levels [13].

Among 100 randomly selected women at CHR, baseline total testosterone concentrations prior to DHEA supplementation were significantly higher than those of NYU patients; 21.7 ng/dL (95% CI, 19.6 - 24,2; P < 0.001). After DHEA supplementation total testosterone concentrations among CHR patients further increased to 32.1 ng/dL (95% CI, 28.6 - 36.1; P < 0.001).

### Embryo ploidy

NYU patients produced an average of 1.51 ± 1.81 euploid embryos and 3.63 ± 2.93 aneuploid embryos. We calculated a euploid-index by dividing the total number of euploid embryos by the total number of embryos biopsied (euploid + aneuploid + no diagnosis). The mean value of this index was 0.29 ± 0.29. We then performed a cluster analysis, subdividing the sample into two groups, based on their evidence of ovarian reserve (day-2 FSH, AMH and units of gonadotropins used) and androgen profiles.

**Figure 1:**
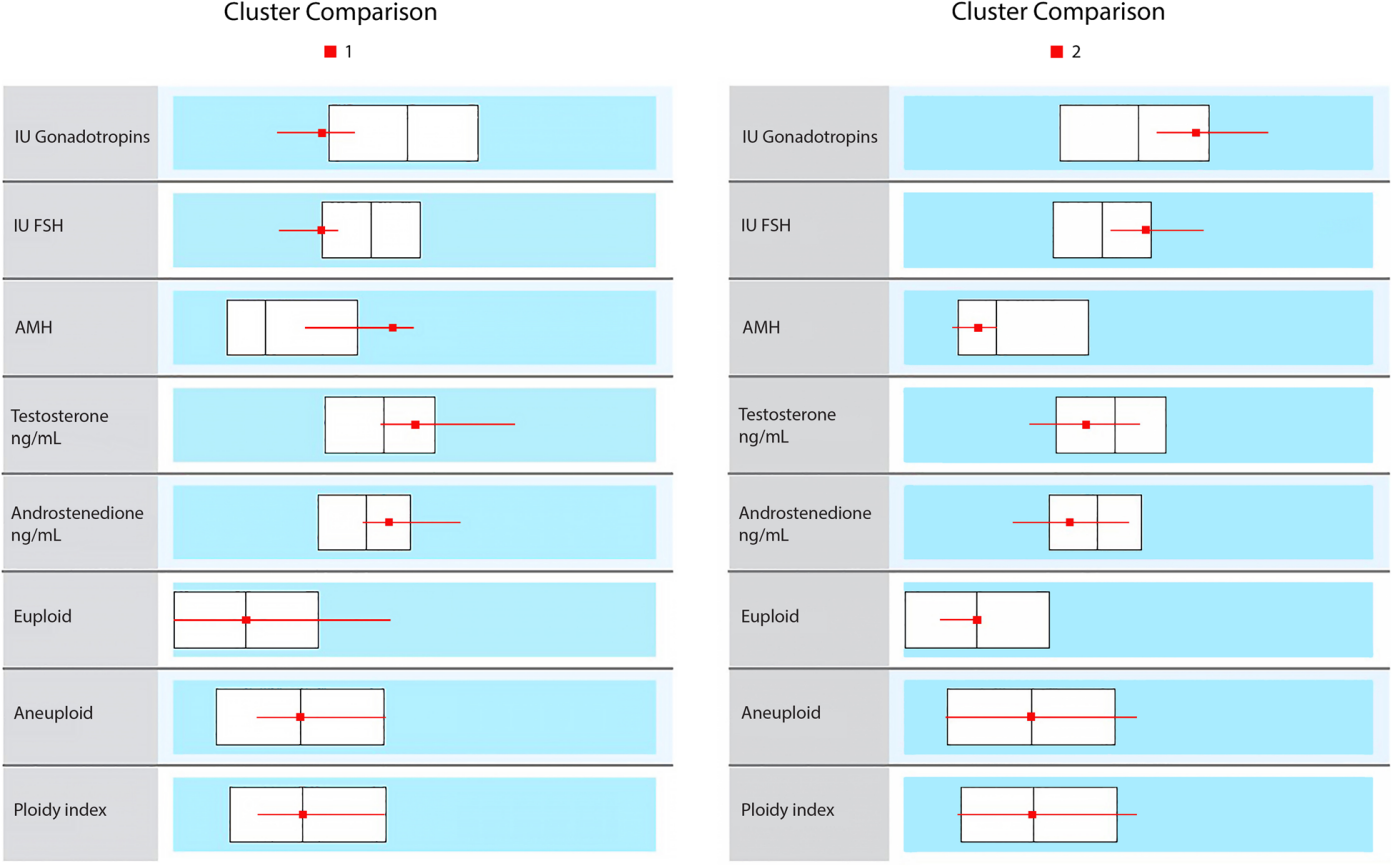
Cluster analysis for NYU study group. This figure demonstrates the cluster analysis of the NYU study group: Cluster 1; Cluster 2; The white boxes represent distributions for the whole NYU study group of patients, while the horizontal lines define the characteristics of patients in the two clusters. Cluster 1 presents women with relatively low gonadotropin dosage requirements of hMG and FSH. As expected, their AMH, testosterone and Androstenedion levels are above median; yet, ploidy is exactly at median. Cluster 2 presents the opposite patient phenotype, with required gonadotropin dosages being high, AMH and androgens low; yet, ploidy again remains at median. The cluster analysis, therefore, demonstrates the expected associations between LFOR, high gonadotropin use and relative hypoandrogenism vs. normal FOR, with low gonadotropin use and normal androgen levels.

As expected based on earlier reports [9], the group with lower ovarian reserve markers also had lower androgen concentrations, while those with better ovarian reserve had higher concentrations of androgens. The euploid-index however remained unchanged for both groups (Figure 1).

Further verifying our model using linear regression, we confirmed that the number of euploid embryos per patients was associated with the number of aneuploid embryos per patient (Beta 0.185, t 1.7; P = 0.09), though this relationship did not reach significance.

Adding total number of fertilized oocytes to the model made the relationship between euploid and aneuploid embryos inverse (Beta −0.437, t −3.18, P = 0.002), and allowed it to reach significance. This model reflects that, after adjusting for numbers of oocytes biopsied, as euploid embryo numbers increase, the number of aneuploid embryos must decrease.

Adding further individual factors to the model allows for the estimation of independent effects of each factor on the model. Adding age to the model, thus, decreased the significance of other factors as predictors of number of euploid embryos because age is also highly correlated with other factors included in the model.

Adding androgen concentrations, either individually or combined, however, did not affect the model in any way, confirming results in above described cluster analysis that androgen concentrations, in ranges found in the NYU dataset, were not associated with the number of euploid embryos.

## Discussion

We in this study attempted to investigate whether androgen levels statistically related to embryo aneuploidy. Obtained results were quite remarkable, though not as expected: In contrast to expectations, multiple regression models failed to demonstrate an association between concentrations of any of the investigated androgens and embryo aneuploidy, whether adjusted for age or not.

This collaboration between two fertility centers in New York City is the first systemic evaluation of associations of androgen concentrations with aneuploidy in an unselected group of infertile women, undergoing IVF and PGS at one New York City-based IVF center (NYU). Patient selection was unbiased because collaborators from the NYU Medical Centers at random selected frozen-stored patient samples from a larger patient pool, and investigators at CHR analyzed samples from a control group of age and by IVF cycle timing matched group of infertility patients.

Androgen analyses in samples included in this study were performed using validated mass spectrometry-based methods of commercial reference laboratory methodology as now recommended for scientific investigations [15], and proven to provide the most specific measurements of endogenous steroids [12,14]. All samples in this study were assayed in blinded fashion, and codes were only broken after results had been obtained.

Reported aneuploidy rates were then statistically associated with patients’ androgen concentrations, first in multiple regression models and later, once regression analyses did not demonstrate statistical associations, by cluster analyses.

Cluster analysis (Figure 1), however, revealed fully expected associations between gonadotropin dosage utilized in IVF cycles and FOR, as defined by FSH and AMH levels. Indeed, it also confirmed previously reported observation of low androgen concentrations (in this case total testosterone and Androstenedion) with LFOR, as assessed by FSH and AMH [9]. Embryo ploidy rates, however, remained steady at exactly median, whether women had lower or higher androgen concentrations or lower or higher FOR by FSH and AMH levels (Figure 1).

These unexpected findings, of course, demand explanation. Only two possible explanations come to mind: In the literature reported associations between LFOR and increased aneuploidy [4,5] and of increased aneuploidy with low androgen concentrations [7,8] are incorrect. While a possibility, we consider this a less likely explanation.

This leaves as the only possible remaining explanation that here-investigated NYU patients, after all, in a subliminal way were selected. In this context it is important to recall that while these patients were random infertility patients, who underwent IVF and PGS at NYU, most of them had previously failed IVF cycles without utilization of PGS. They, thus, indeed represented a negatively selected patient population with relative poor outcome chances. Such patients usually exhibit LFOR, and with this diagnosis they can be expected to demonstrate low androgen concentrations [9].

Here investigated NYU patients, indeed, demonstrated unusually low androgen levels, as this study demonstrated. They, however, in a somewhat contradictory presentation, also still demonstrated based on AMH, FSH levels and oocyte yields surprisingly good FOR, considering their rather ages (Table 1).

Such patients are currently under investigation at CHR, with preliminary evidence suggesting that they represent women with a lean polycystic ovary syndrome (PCOS) – like ovarian phenotype at young ages, at older ages characteristically, however, presenting with severe hypoandrogenemia; yet, as here observed in NYU patients, still surprisingly good FOR (reflected in AMH and oocyte yield) considering their ages (Gleicher N, Kushnir VA, Barad DH, unpublished observations).

Comparing DHEA, Androstenedion and testosterone concentrations in these NYU patients with a similarly selected group of infertility patients during the same time period undergoing IVF at CHR, the NYU group demonstrated significantly lower androgen concentrations than the CHR group, a difference which even further expanded after CHR patients were supplemented with DHEA, currently at CHR a routine practice in women with LFOR [16].

Published evidence of an association of androgen concentrations with embryo aneuploidy rates in two so far published studies, however, comes exclusively from LFOR patients supplemented with DHEA [7,8]. If one were to assume that the results of these two studies are valid, the only possible explanation for here reported absence of correlation between androgen levels and embryo aneuploidy would be that such a quantitative association only exists above a certain minimum androgen levels, which DHEA-supplemented women, of course, would always exceed.

This, of course, is presently only a hypothesis but a hypothesis, which can be confirmed with relative ease by repeating here reported study in an infertile population with either normal androgen levels or in an infertile population with very low androgen levels (like here investigated NYU patients) who, however, prior to IVF cycle start receive androgen supplementation to raise their testosterone levels into normal range.

Our here presented study, therefore, requires a significant follow up effort before final statements about the effects of androgen levels on embryo ploidy can be made. What, however, can be stated quite unequivocally based on this study is that in infertile women with severe hypoandrogenemia androgen levels do not determine aneuploidy.

Should further studies confirm our hypothesis of a threshold level for androgens below which androgen effects on embryo ploidy are lost, definition of minimal androgen concentrations (i.e., a certain threshold) above which aneuploidy rates would decline in age-specific degrees would open potential new therapeutic options for improving IVF pregnancy chances and reducing miscarriage risks. Consistent with such a prospect miscarriage rates after DHEA supplementation start declining only after approximately age 35 [8].

## Abbreviations

aCGH: array comparative genomic hybridization
AMH: anti-Müllerian hormone, CHR, Center for Human Reproduction
DHEA: dehydroepiandrosterone
E2: estradiol
FOR: functional ovarian reserve
FSH: follicle stimulating hormone
IVF: in vitro fertilization
GnRH: gonadotropin releasing hormone
hCG: human chorionic gonadotropin
hMG: human menopausal gonadotropin
LFOR: low functional ovarian reserve
IVF: in vitro fertilization
NYU: New York University
PGS: preimplantation genetic screening
POA: premature ovarian aging
2-PN: two pronuclear
